# Risk factors, logistic model, and vulnerability mapping of lumpy skin disease in livestock at the farm level in Indragiri Hulu District, Riau Province, Indonesia, in 2022

**DOI:** 10.14202/vetworld.2023.2071-2079

**Published:** 2023-10-07

**Authors:** Tri Susanti, Heru Susetya, Prima Widayani, Yul Fitria, Gigih Tri Pambudi

**Affiliations:** 1 Department of Epidemiology and Veterinary Public Health, Graduate Student of Veterinary Science, Faculty of Veterinary Medicine, Gadjah Mada University, Yogyakarta, Indonesia; 2 Disease Investigation Centre of Bukittinggi, Bukittinggi, Indonesia; 3 Department of Epidemiology and Veterinary Public Health, Faculty of Veterinary Medicine, Gadjah Mada University, Yogyakarta, Indonesia; 4 Department of Geographical Information Science, Faculty of Geography, Gadjah Mada University, Yogyakarta, Indonesia

**Keywords:** Indragiri hulu, lumpy skin disease, risk factors, vulnerability map

## Abstract

**Background and Aim:**

Lumpy skin disease (LSD) is an emerging epidemic in livestock in Indonesia. It was first reported in the Indragiri Hulu Regency of Riau Province, which has more cases than the surrounding regencies. This study aimed to identify the risk factors and generate a logistic regression model and vulnerability map of LSD in the Indragiri Hulu Regency.

**Materials and Methods:**

We used a structured questionnaire to interview the case and control farm owners to evaluate the risk factors. We evaluated 244 samples, consisting of 122 case and control farm samples each. At the cattle farm level, the risk factor data related to LSD were analyzed using descriptive statistics, bivariate analysis with Chi-square, and odds ratio, while the logistic model was derived using multivariate logistic regression analysis. Using variables, such as the number of cases and risk factor variables included in the model logistic, and the temperature, humidity, and rainfall data from the Meteorology, Climatology, and Geophysical Agency, we analyzed the vulnerability map of LSD in the regency using scoring, weighting, and overlay methods.

**Results:**

Ten significant risk factors were associated with LSD occurrence. The LSD model obtained from the logistic regression analysis was LSD (Y) = −3.92095 + 1.13107 (number of cattle >3) + 1.50070 (grazing cattle together with other farmers’ cattle) + 1.03500 (poor management of farm waste/dirt) + 2.49242 (presence of livestock collectors/traders near the farm location) + 1.40543 (introduction of new livestock) + 2.15196 (lack of vector control measures on the farm). The LSD vulnerability map indicated that the villages with high vulnerability levels were Rantau Bakung, Kuantan Babu, and Sungai Lala in the Rengat Barat, Rengat, and Sungai Lala subdistricts, respectively.

**Conclusion:**

We found 10 significant risk factors associated with LSD occurrence. The LSD model included the number of cattle (>3), cograzing with other farmers’ cattle, poor management of farm waste/dirt, the presence of livestock collectors/traders near the farm, introduction of new livestock, and lack of vector control measures on the farm. The LSD vulnerability map indicated that villages with high vulnerability levels included Rantau Bakung in the Rengat Barat subdistrict, Kuantan Babu in the Rengat subdistrict, and Sungai Lala in the Sungai Lala subdistrict.

## Introduction

Lumpy skin disease (LSD), also known as nodular dermatitis or cattle pox, is caused by a virus from the Poxviridae family and the Capripoxvirus genus [1]. Cattle, buffalo, and some wild ruminants, such as giraffes, impalas, and musk deer, are susceptible to LSD [2, 3]. The disease has high morbidity and mortality rates, ranging from 5%–45% to 1%–3%, respectively [4, 5]. Although LSD is not a zoonotic disease, it causes significant economic losses [6]. Infected animals undergo weight loss, carcass damage, reduced milk production, mastitis, infertility, and abortion [7, 8]. Treatment and recovery from LSD can take several months. Even if the animal recovers, the disease lesions cause permanent scars, decreasing skin, and fur quality [1].

Lumpy skin disease is a new epidemic disease in livestock in Indonesia. The first positive case of LSD was reported in Indragiri Hulu district, Riau Province, and was confirmed through PCR testing by Balai Veteriner Bukittinggi (Letter No: 15001/PK.310/F4B.1/02/2022) and Balai Besar Veteriner Bogor (Letter No: B-201/PK.310/H.5.1/2/2022) in February 2022. Riau Province was declared as an LSD outbreak area based on the Minister of Agriculture’s decree no 242/KPTS/PK.320/M/3/2022 in March 2022. Based on the clinical diagnosis of LSD conducted by field animal health officers in Riau Province from January 1 to July 31, 2022, the highest number of LSD cases was found in Indragiri Hulu district, with 322 cases from 169 farms, with a proportion of 0.79% [9]. The risk factors for LSD can be related to animals, husbandry systems, and the environment [10, 11]. Although all breeds, ages, and sexes of cattle are susceptible to LSD, European breeds (exotic breeds) are at higher risk than local cattle [11]. The husbandry system and agroclimatic conditions (temperature/humidity/rainfall) are considered the primary risk factors behind the spread of LSD in any region [12]. The spread of the Capripox virus between countries or regions is mainly related to the introduction of new livestock or illegal animal transportation [10, 11, 13, 14]. Lumpy skin disease virus has also been reported without any new livestock introduction or the introduction of new animals into infected herds. This transmission is likely to occur because of the role of vectors or other blood-sucking arthropods that can fly and are wind-borne [15].

As the traditional cattle farming system in the Indragiri Hulu district has low biosecurity measures, the presence of LSD in a farm significantly increases the risk of transmission to neighboring farms. In addition, this district is strategically located as it is crossed by the East Sumatra arterial road, which might be used as a transportation route for livestock between districts and provinces. This increases the threat of LSD transmission in this district and, subsequently, into the surrounding districts.

Therefore, this study aimed to identify the risk factors and to create a logistic model of LSD at the farm level by capturing LSD risk factors and vulnerability mapping. Our results might serve as a reference for determining prevention and control strategies and priority scales for LSD in this district and surrounding districts.

## Materials and Methods

### Ethical approval and Informed consent

The study was approved by Animal Ethics Committee of the Faculty of Veterinary Medicine, Gadjah Mada University, Yogyakarta, Indonesia. The certificate of ethical permission is numbered 020/EC-FKH/Eks/2023, dated February 3, 2023. The verbal consent \was obtained from the participants before the interview.

### Study period and location

**Figure-1:**
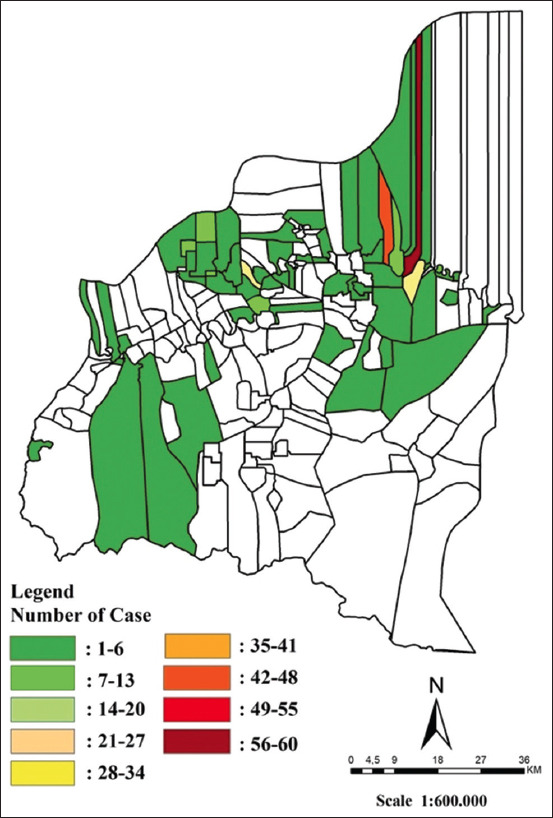
Study area and distribution map of lumpy skin disease case January–July 2022 in Indragiri Hulu District (Source: Map Prepared by the corresponding author).

This study was conducted from February to April 2023 in Indragiri Hulu district, Riau Province. The study area consisted of villages where LSD cases were detected in this district from January 1 to July 31, 2022 (Figure-1).

### Study design and sample size

This study employed a case-control study design to identify significant risk factors that increase the incidence of LSD, thereby obtaining a logistic model and vulnerability map for LSD in the Indragiri Hulu district.

The sample size for the case-control study was determined as follows [16]:


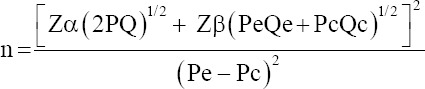


Where N is the sample size, Zα is the value of the type I error (1.96) with 5% error rate, Zβ is the value of the type II error (−0.84) with 20% error rate, Pe is the estimated outcome for the exposed group, Qe is 1-Pe, Pc is the estimated outcome for the unexposed group, Qc is 1−Pc, P is (Pe + Pc)/2, and Q is 1−P; P is (Pe + Pc)/2.

Lumpy skin disease prevalence and risk factors were estimated using LSD seroprevalence of 19.5% and OR value of 2.216 reported in Egypt [17] as follows:

Pe estimation = 0.349, where Pe = (OR × Pc)/[OR × Pc + (1−Pc)].

Qe = 1−Pe: 1−0.349 = 0.651

Pc estimation = 0.195

Qc = 1−Pc: 1−0.195 = 0.805

P = (Pe + Pc)/2 = (0.349 + 0.195)/2 = 0.234


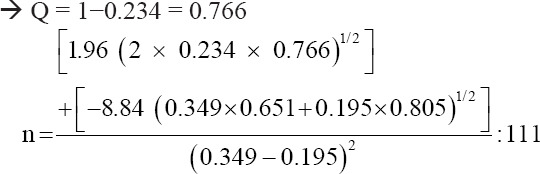


Thus, the minimum sample size required was 111.

Based on this, we need a minimum of 111 cases and control farms, each with a 1:1 ratio. The determination of the sample size from each district or village is proportional. Case farms were randomly selected from 169 LSD case farms identified using the LSD diagnosis data from the Department of Animal Husbandry and Fisheries of Indragiri Hulu Regency from LSD case monitoring activities by veterinarians/animal health officers of the Regency, which were compiled from January 1 to July 31, 2022. Based on proportional farm sample calculations for each village (rounded up), we sampled 122 LSD case and control farms each.

### Sampling criteria

The case and control cattle farms were selected based on the inclusion and exclusion criteria. The former included farms with a definitive diagnosis of LSD confirmed by continuously monitoring the disease progression by local animal health officers from January 1 to July 31, 2022. Lumpy skin disease was diagnosed based on typical clinical symptoms, such as skin nodules (1–5 cm in diameter), elevated body temperature (40°C–41°C), and decreased appetite. Meanwhile, the control farms are those that do not have a history of LSD cases and are matched based on the location closest to the case farms. The exclusion criteria comprised case farms that did not have case monitoring data and an unclear address, and farmers were not willing to be interviewed/observed or were not domiciled in the Indragiri Hulu district.

### Data collection

We used primary and secondary data for this study. Primary data were obtained through field observations using a questionnaire to determine the risk factors associated with the incidence of LSD and data plotting coordinates (x, y) of case farm locations using Global Positioning System instruments. Secondary data were based on the clinical diagnosis of LSD through LSD monitoring by veterinarians/animal health officers in Riau Province (Department of Animal Husbandry and Animal Health of Riau province), which were recapitulated from January 1 to July 31, 2022. Other secondary data included weather data (temperature, rain, and humidity) from January 1 to July 31, obtained from the Meteorology, Climatology, and Geophysics Agency.

### Questionnaire

The data on the risk factors for LSD at the farm level were obtained by interviewing the farmers using a questionnaire that was validated and tested for reliability using Statistical Package for the Social Sciences for Windows version 25 (IBM Corp., NY, USA). To conduct this test, a pretest questionnaire was administered to 20 respondents to ensure data normality, which is crucial for statistical calculations. Validity and reliability were measured using the SPSS Person correlation and reliability analysis instruments. The questionnaire items were deemed valid when the p-value was <0.05 and highly reliable when the Cronbach’s alpha value was >0.6.

### Statistical analysis

Descriptive, bivariate, and multivariate analyses were performed using Statistix 9.1 (https://statistix.informer.com/9.1/), while vulnerability mapping analyses were conducted using ESRI ArcGIS Desktop (ArcMap) version 10.8.2 software (https://tinyurl.com/2amnzhw5). Descriptive analysis was conducted for each variable by presenting the frequency distribution of the variables under investigation. In bivariate analysis, the Chi-square and p-values were calculated to determine whether the effect of the risk factors on LSD occurrence is significant or not, with a significance level of α = 0.05 and a table χ^2^of 3.85. The risk factors were deemed significant if the calculated χ^2^ value is greater than the table χ^2^ value and p < α. Furthermore, the odds ratio (OR) was calculated for the significant risk factors to determine the strength of the association between the risk factor and the disease. Subsequently, we performed multivariate analysis using logistic regression analysis with a significance level of p = 0.05 and a confidence level of 95% to obtain a prediction model for LSD incidence in the Indragiri Hulu district, which was formulated as Y = α + β1X1 + β2X2 +…. + βnXn + e [18, 19]. Vulnerability mapping analysis was performed using scoring, weighting, and overlay methods. The variables used include the number of LSD cases, risk factors included in the logistic model, and weather data (temperature, humidity, and rainfall) in the Indragiri Hulu district from secondary data sources from Meteorology, Climatology, and Geophysical Agency for the period from January to July 2022. For the risk factors of the logistic model, scoring and weighting are determined based on the coefficients of the LSD Logistic model variables, while for temperature, rainfall, and humidity, scoring, and weighting are determined based on the literature studies that influence vector development. The vulnerability level is calculated based on the difference between the maximum and minimum values of the total score of all LSD risk factors [20]. We tested the vulnerability map model to assess its accuracy using an LSD case distribution map, which was plotted with its location coordinates on the map. The percentage of case distribution was examined based on the location of these vulnerability zones [21].

## Results

We interviewed 244 respondents or farmers, one each from 122 case farms and 122 control farms, to investigate the risk factors of LSD at the farm level in the Indragiri Hulu district. Tables-1–3 show the results of univariate, bivariate, and multivariate analyses.

**Table-1:** Univariat and bivariate analysis of the LSD risk factor at the farm level.

No.	Variable	Category	Univariate				Bivariate		
	
			LSD on farm				p-value	Chi-square	OR

			Yes	No	Total	%			
1	Education	<Senior high school	69	63	132	54.1	0.441	0.590	
		≥Senior high school	53	59	112	45.9			
2	Participate in livestock counseling in the last 2 years	No	107	99	206	84.4	0.158	1.990	
		Yes	15	23	38	15.6			
3	Age	>64 years	8	3	11	4.5	0.123	2.380	
		15–64 years	114	119	233	95.5			
4	Take care of the cattle	≤10 years	86	63	149	61.1	0.003	9.120	2.24
		>10 years	36	59	95	38.9			
	Farm management								
5	Number of cattle	>3	97	50	147	60.2	0.000	37.80	5.59
		≤3	25	72	97	39.8			
6	Farm location	Low land	103	93	196	80.3	0.100	2.700	
		High Land	19	29	48	19.7			
7	Intensive farming system	Yes	52	79	131	53.7	0.001	12.020	0.41
		No	70	43	113	46.3			
8	Semi intensive farming system	Yes	49	40	89	36.5	0.231	1.430	
		No	73	82	155	63.5			
9	Extensive farming system (Pastoral)	Yes	21	3	24	9.8	0.001	14.970	8.25
		No	101	119	220	90.2			
10	Farm location near the river. lake	Yes	62	30	92	37.8	0.000	17.870	3.17
		No	60	92	152	62.2			
11	Grazing cattle together with other farmers cattle	Yes	66	35	101	41.4	0.000	14.390	2.93
		No	56	87	143	58.6			
12	Animal species on farm	Mix	19	14	33	13.5	0.349	0.880	
		Cattle only	103	108	211	86.5			
13	Manage of waste (manure/leftover feed)	Not well managed	107	74	181	74.2	0.000	23.3	4.63
		Well managed	15	48	63	25.8			
14	Vector control measure on the farm	No	99	40	139	57.0	0.000	48.27	8.82
		Yes	23	82	105	43.0			
15	Presence of livestock collectors/traders near the farm location	Yes	42	4	46	18.9	0.000	38.68	15.5
		No	80	118	198	81.1			
16	Introduction of the new cattle in farm	Yes	26	6	32	13.1	0.000	14.39	3.59
		No	116	96	212	86.9			
17	location of the farm close to cattle traffic lanes between villages/districts/provinces	Yes	61	49	110	45.1	0.123	2.38	
		No	61	73	134	54.9			
18	Fence in farm	No	91	86	177	72.5	0.473	0.51	
		Yes	31	36	67	27.4			
19	There is trucks or animal/goods transport vehicles enter the farm	Yes	24	25	49	20.1	0.873	0.03	
		No	98	97	195	79.9			
20	Vaccination	No	122	117	239	98.0	0.024	5.1	
		Yes	0	5	5	2.0			

LSD=Lumpy skin disease, OR=Odds ratio

**Table-2:** Logistic regression analysis model at farm level in Indragiri Hulu district.

Predictor variables	Koefisien	SE	Koefisien/SE	Nilai P
Constant	−3.92095	0.61609	−0.36	0.0000
Number of cattle > 3	1.13107	0.37602	3.01	0.0026
Grazing cattle together with other farmers cattle	1.5007	0.39612	3.79	0.0002
Poor management of farm waste/dirt	1.035	0.49682	2.08	0.0372
Presence of livestock collectors/traders near the farm location	2.49242	0.60933	4.09	0.0000
Introduction of new livestock	1.40543	0.5963	2.36	0.0184
Lack of vector control measures on the farm	2.15196	0.39211	5.49	0.0000
Deviance	197.1			
p-value	0.9723			
Degree of freedom	237			

**Table-3:** The odds ratio value in the LSD model at farm level in Indragiri Hulu district.

Predictor variables	95%CI	Odds ratio	Upper limit

	Lower limit		
Number of cattle > 3	1.48	3.1	6.48
Grazing cattle together with other farmers cattle	2.06	4.48	9.75
Poor management of farm waste/dirt	1.06	2.82	7.45
Presence of livestock collectors/traders near the farm location	3.66	12.09	39.91
Introduction of new livestock	1.27	4.08	13.12
Lack of vector control measures on the farm	3.99	8.6	18.55

LSD=Lumpy skin disease, CI=Confidence interval

### Description of cattle farms in Indragiri Hulu regency

The sampled cattle farms in Indragiri Hulu Regency are still traditional (backyard). The average age of the farmers ranged between 15 and 64 years (95.5%) and the minimum education level was high school (45.9%). Most of them (84.4%) did not attend any livestock extension activities in the past 2 years, and about 61.1% have the same or <10 years of experience in cattle farming. The livestock management system in this regency is divided into three forms: Intensive, where the cattle are kept in a barn (53.7%); semi-intensive, where the cattle are kept in a barn at night and released to pasture during the day (36.5%); and extensive, where the cattle are only released to graze in a grazing area or plantation (9.8%).

### Bivariate analysis of LSD risk factors at the farming level

Table-1 shows the bivariate analysis of LSD risk factors in livestock farming in the Indragiri Hulu district. The significant risk factors are the duration of livestock rearing by the farmer, which was similar or below 10 years (p = 0.003, OR = 2.24), the number of livestock (n > 3) (p = 0.000, OR = 5.59), an intensive (p = 0.001, OR = 0.41) or extensive (p = 0.001, OR = 8.25) systems of animal husbandry, the presence of a river or lake around the cage/grazing area (p = 0.000, OR = 3.17), grazing the livestock together with other farmers’ livestock (p = 0.000, OR = 2.93), poorly managed feed and manure waste (p = 0.000, OR = 4.63), lack of vector control on the farm (p = 0.000, OR = 8.82), the presence of livestock collectors/traders near the farm location (p = 0.000, OR = 15.5), and the introduction of new livestock (p = 0.000, OR = 3.59). Furthermore, the risk factors that were not significantly associated with the occurrence of LSD in livestock at Indragiri Hulu district were the education level of the livestock owners not reaching beyond elementary school (p = 0.441), not attending livestock extension programs at least 2 years prior (p = 0.158), livestock owners not being in the productive age range of 15–64 years (p = 0.123), location of livestock in low-lying areas (p = 0.1), semi-intensive livestock management system (p = 0.231), raising more than one species (mixed) (p = 0.349), livestock located near roads or traffic (p = 0.13), livestock location separated by fences (p = 0.473), trucks entering the livestock area (p = 0.873), and vaccination (p = 0.024).

### Multivariate analysis of LSD risk factors in livestock farming

Table-2 shows the multivariate analysis results obtained from logistic regression analysis. The LSD model from this analysis is LSD (Y) = −3.92095 + 1.13107 (number of livestock >3) + 1.5007 (livestock grazed together with other farmers’ livestock) + 1.035 (improper management of livestock waste) + 2.49242 (Proximity to livestock traders/collectors) + 1.40543 (Introduction of new livestock) + 2.15196 (Absence of vector control measures on the farm).

The results of logistic regression analysis of the risk factors of LSD in livestock farms (Tables-2 and 3) indicate that the factors contributing to the increased occurrence of LSD in livestock farms are: Having more than three heads of livestock (β = 1.13107, OR = 3.1), grazing livestock along with those from other farms (β = 1.5007, OR = 4.48), poorly managed waste (manure and leftover feed) (β = 1.035, OR = 2.82), the presence of livestock collectors near the farm (β = 2.49242, OR = 12.09), the introduction of new animals (β = 1.40543, OR = 4.08), and lack of vector control measures in the farm (β = 2.15196, OR = 8.6). The model obtained was relatively accurate as it passed the goodness-of-fit (Hosmer-Lemeshow) test with a sensitivity of 86.06% and specificity of 73.77%.

### Vulnerability mapping analysis

Based on the logistic regression model, the risk factors that increase LSD occurrence are the number of livestock with more than three heads, grazing together with livestock from other farms, poorly managed waste (manure and feed residues), the presence of livestock collectors near the farm, introduction of new livestock, and the absence of vector control activities in the farm. Table-4 indicates the scores for each risk factor.

**Table-4:** Vulnerability of LSD variable based on case and risk factor of farm level.

Predictor variables	Category	Coefficient (weighting)	Score
LSD case	High (31–60)		2
	Low (1–30)		1
Number of cattle	>3	1.13107	2
	≤3		1
Grazing cattle together with other farmers cattle	Yes	1.5007	2
	No		1
Manage of waste (manure/leftover feed)	Not well managed	1.035	2
	Well managed		1
Presence of livestock collectors/traders near the farm location	Yes	2.49242	2
	No		1
Introduction of new livestock	Yes	1.40543	2
	No		1
Vector control measure on the farm	No	2.15196	2
	Yes		1

LSD=Lumpy skin disease

Based on data from the Meteorology, Climatology, and Geophysical Agency, the range of temperature, humidity, and rainfall in Indragiri Hulu district from January 1 to July 31, 2022, is 26.31°C–27.18°C, 83.83%–84.88%, and 233.534–253.027 mm, respectively (Table-5) [22–25]. The temperature and humidity variables in all areas of Indragiri Hulu district are in score 3 (High), while rainfall is in score 2 (Middle), indicating that all areas in the Indragiri Hulu district have the same vulnerability level in terms of temperature, humidity, and rainfall variables.

**Table-5:** Vulnerability score of LSD variables based on temperature, humidity, and rainfall.

Predictor variables	Range	Score	Reference
Temperature (C)	<25	1	[22–25]
	25–27	3	
	>27	2	
Humidity (%)	<76.5	1	[23, 24]
	80.5–89	3	
	>89	2	
Rainfall (mm)	<275	2	[25]
	275–375	3	
	>375	1	

LSD=Lumpy skin disease

**Figure-2:**
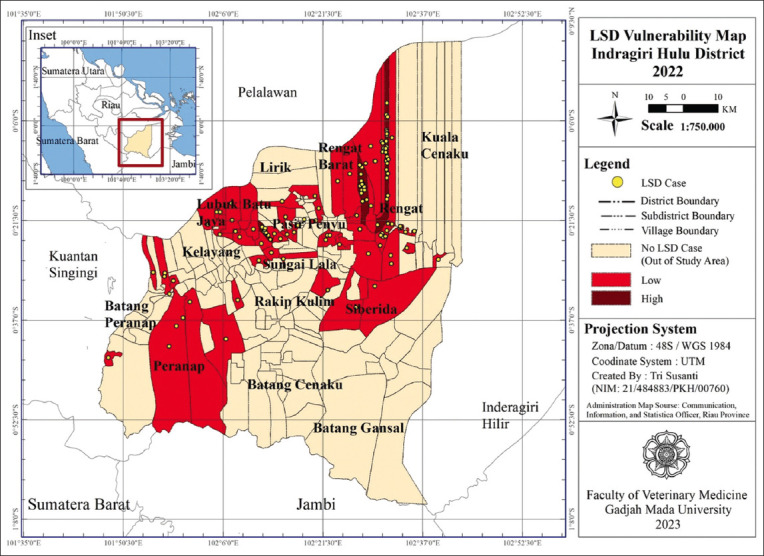
Vulnerability Map of Lumpy Skin Disease in Indragiri Hulu District, 2022 (Source: Map Prepared by the corresponding author).

The vulnerability scores were calculated based on the difference between the maximum and minimum values of the total scores of all LSD risk factors (Table-6) and the vulnerability map was obtained (Figure-2). The LSD vulnerability map 2022 in this district shows that the most vulnerable areas were the villages of Rantau Bakung in the West Rengat subdistrict, Kuantan Babu in the Rengat subdistrict, and Sungai Lala in the Sungai Lala subdistrict. Plotting the coordinates of the 122 cases shows that the highest number of cases was spread across these three villages, namely, 22 cases (18.3%) from Kuantan Babu village, 14 cases (11.47%) from Rantau Bakung village, and 10 cases (8.1%) from Sungai Lala village.

**Table-6:** Variable scoring result.

No.	Total score	Vulnerability
1	67–865	Low
2	866–1799	High

## Discussion

The significant risk factors for LSD in livestock farms based on bivariate analysis were <10 years of farming experience, more than three heads of livestock, grazing, or releasing livestock together with other farmers, poor management of waste (manure and leftover feed), the proximity of livestock collectors to the farm location, introduction of new livestock, and lack of vector control activities in the farm. Less than 10 years of farming experience had a significance value of 0.003 and OR of 2.24, indicating that livestock farming conducted for <10 years has a 2.24 times higher risk for LSD than those with more than 10 years of experience. The duration of livestock farming reflects the farmer’s experience in the field. Poor farming experience might suggest that the skills and livestock management practices of the farmer are still insufficient, thus making them inexperienced in determining necessary actions to prevent and control diseases on the farm, specifically for LSD [26].

The significant risk factor for the occurrence of LSD in livestock farms based on bivariate analysis is the number of livestock raised on the farm, which is more than three. This factor has a significant value of p = 0.000 and an OR of 5.59, indicating that farms with more than three livestock are 5.6 times more likely to experience LSD than farms with three or fewer animals. This finding is consistent with the previous research by Kiplagat *et al*. [11], which showed an association between livestock over three and LSD. In addition, another study Hailu *et al*. [14] found that livestock over 12 are correlated with LSD. The number of livestock most likely reflects the farmers’ ability to provide adequate nutrition and attention to their animals’ health.

The significant risk factors affecting the incidence of LSD are the intensive (p = 0.001, OR = 0.41) and extensive (p = 0.001, OR = 8.25) livestock farming systems, suggesting a negative association of intensive livestock farming systems with LSD incidence (by 0.41). In other words, intensive farming systems can prevent LSD occurrence by 0.41 times compared to semi-intensive and extensive farming systems. Furthermore, extensive livestock farming systems are positively associated with LSD incidence, which is 8.25 times higher than that of intensive and semi-intensive farming systems. The increase in LSD transmission in extensive farming systems might be because the animals only graze in open fields without any permanent enclosures. In open fields, the animals can become infected through communal food and water sources that may have already been contaminated by the LSD virus [17]. Moreover, in extensively grazed animals, the provision of animal feed relies solely on food availability in the grazing area [27]. Under certain conditions, such as the dry season, the limited amount of green forage might result in insufficient nutrition for the livestock, making them more susceptible to diseases. In an intensive farming system, the nutritional needs of the livestock are carefully considered and met accordingly. Some farmers even provide additional feed to improve the nutrition of their livestock, thereby enhancing their immune functions and making them more disease-resistant [28].

The risk factor of the presence of a river or lake near a farm was significantly associated with LSD incidence (p = 0.000, OR = 3.17), suggesting that the incidence of LSD in farms located near rivers or lakes is 3.17 times higher than that in farms located far from or without rivers or lakes nearby. This is also probably related to the abundance of vectors on the farm. According to Tuppurainen and Oura [13], farms near river deltas and basins, and areas with standing water, impact the abundance of vectors that spread the LSD virus.

Another significant risk factor associated with LSD occurrence is the practice of grazing animals and those owned by other farmers (p = 0.001 and OR = 2.5). This suggests that LSD is 2.5 times more likely to occur on farms where animals are grazed together with those owned by other farmers than on farms where animals are not grazed together with those owned by other farmers. This finding is consistent with the study by Selim *et al*. [17], who states that grazing animals together can increase the likelihood of contact and transmission of infection. Communal livestock grazing also increased the risk of LSD transmission through feed or communal drinking water that is already contaminated with LSD virus from other infected animals [10, 29]. According to Hailu *et al*. [14], pastoral livestock farming systems are twice as susceptible to LSD infection, due to the grazing or communal water sources that are contaminated with LSD from other infected livestock.

The farms that do not manage animal waste properly are significantly associated with LSD incidence (p = 0.000) with an OR of 4.63, suggesting that farms that do not manage animal waste properly are 4.63 times more likely to contract LSD than those that manage animal waste properly. This might be related to vector abundance on the farm. Vectors prefer wet areas with scattered or piled feces on farms [30]. Livestock waste, such as feces, leftover feed, and urine, can be treated separately. Leftover feed can be burned, urine can be collected and used as fertilizer, and feces can be used for compost. Feces should not be piled up in animal pens or the farm area but should be placed in a covered area far away from the livestock. Proper waste management can reduce the breeding sites for vectors on the farm.

The vector control on farms has a significant association (0.000) with an OR of 8.8, indicating that farms with no vector control are 8.8 times more predisposed to LSD infections than those with adequate vector control. The vector control activities in this district include fumigation or burning of waste in the farm area (traditional method) to reduce mosquitoes and flies, spraying insecticides to control flies and mosquitoes, and providing anti-tick or anti-flea medication to livestock. As LSD is transmitted by blood-sucking arthropods, such as flies, mosquitoes, and ticks, the abundance of vectors on a livestock farm can increase the risk of LSD occurrence [15]. Therefore, routine vector control activities are needed in livestock farms while considering environmental health, which means avoiding the excessive use of insecticides.

The association of livestock farms with animal collectors nearby (<5 km) with LSD incidence was 15.49 times higher (OR 15.49 and p = 0.000) than other risk factors. This is likely related to the activities of animal collectors or traders who often transport livestock from other areas that may be infected with LSD. This livestock is usually kept in temporary pens before being traded or sold. During this period, these animals can contract and transmit LSD because they are rarely subjected to health examinations. Hence, these animals might be possibly infected with LSD and potentially transmit it to nearby farms, especially through the vectors, such as flies and mosquitoes. According to Gubbins *et al*. [31], LSD transmission mostly occurs within a short distance (<5 km), which can be linked to vector-borne transmission.

Introducing new livestock has an association (0.000) with a strength of association of 3.59. This indicates that LSD incidence in farms with a history of introducing or purchasing new livestock before the incidence is 3.59 times higher than that in farms that do not introduce or purchase new livestock. Several studies have also shown a significant association between LSD incidence and history of introducing new livestock into the population [10, 17, 32]. This may be because the introduced livestock did not undergo prior screening or testing, increasing the likelihood of LSD infection during incubation [14].

Modeling and vulnerability mapping are commonly used in epidemiological analysis. Infectious disease models can be used to predict disease occurrence in a particular area, providing a reference for preventive measures to contain disease spread [33]. Logistic regression analysis can be used to obtain a model of categorical dependent variables, and categorical and continuous independent variables to estimate the probability of an event occurring based on several independent variables [18, 34]. In this study, ten significant risk factors were found to be linked with LSD occurrence (Table-2), of which six were included in the logistic regression model (Table-3) for LSD in the Indragiri Hulu district, which is as follows: (Y) = −3.92095 + 1.13107 (number of livestock >3) + 1.50070 (grazed together with livestock belonging to other farmers) + 1.03500 (poorly managed livestock waste) + 2.49242 (presence of animal collectors/traders near the livestock farm) + 1.40543 (introduction of new livestock) + 2.15196 (lack of vector control activities in the livestock farm).

The vulnerability level forms the basis for determining disaster vulnerability. Mapping vulnerability zones can be used to map the prevalence of diseases, identify the transmission sources, and predict disease occurrence in an area [20]. The LSD vulnerability map 2022 in this district shows that the most vulnerable areas were the villages of Rantau Bakung in the West Rengat subdistrict, Kuantan Babu in the Rengat subdistrict, and Sungai Lala in the Sungai Lala subdistrict. These villages are located in a high vulnerability zone due to the high number of LSD cases compared to other villages, which is also exacerbated by the presence of LSD risk factors, such as having more than 3 heads of livestock, grazing together with other livestock, poor management of waste/dirt, the proximity of collectors to the livestock location, the presence of new livestock inputs, and the absence of vector control/eradication activities in the livestock farms. Meanwhile, temperature, humidity, and rainfall show the same vulnerability for all regions in Indragiri Hulu Regency.

## Conclusion

We identified ten significant risk factors associated with LSD occurrence. Of these, the following six were included in the LSD model: The number of cattle >3, grazing cattle together with other farmers’ cattle, poor management of farm waste/dirt, the presence of livestock collectors/traders near the farm location, introduction of new livestock, and lack of vector control measures on the farm. The LSD vulnerability map indicated that the villages of Rantau Bakung in the Rengat Barat subdistrict, Kuantan Babu in the Rengat subdistrict, and Sungai Lala in the Sungai Lala subdistrict had high vulnerability levels.

## Authors’ Contributions

TS: Designed and conducted the study. TS, HS, and PW: Planned the study and analyzed the results. YF and GTP supervised the study. HS, PW, YF, and GTP: Corrected and reviewed the manuscript. All authors have read, reviewed, and approved the final manuscript.
